# Soluble Forms of Immune Checkpoints and Their Ligands as Potential Biomarkers in the Diagnosis of Recurrent Pregnancy Loss—A Preliminary Study

**DOI:** 10.3390/ijms25010499

**Published:** 2023-12-29

**Authors:** Michał Zych, Aleksander Roszczyk, Filip Dąbrowski, Monika Kniotek, Radosław Zagożdżon

**Affiliations:** 1 Department of Clinical Immunology, Transplantation Institute, Medical University of Warsaw, Nowogrodzka 59, 02-006 Warsaw, Mazovian Voivodeship, Poland; michal.zych27@gmail.com (M.Z.); aleksander.roszczyk@gmail.com (A.R.); radoslaw.zagozdzon@wum.edu.pl (R.Z.); 2 Department of Obstetrics, Perinatology and Neonatology, Center of Postgraduate Medical Education, Ceglowska 80, 01-809 Warsaw, Mazovian Voivodeship, Poland; fil.dabrowski@gmail.com; 3 Club35, Polish Society of Obstetricians and Gynecologists PTGiP, Cybernetyki7F/87, 02-677 Warsaw, Mazovian Voivodeship, Poland; 4 1st Department of Obstetrics and Gynecology, Medical University of Warsaw, Starynkiewicza 1, 02-015 Warsaw, Mazovian Voivodeship, Poland; 5 Department of Immunology, Transplantology, and Internal Diseases, Medical University of Warsaw, Nowogrodzka 59, 02-006 Warsaw, Mazovian Voivodeship, Poland

**Keywords:** soluble immune checkpoints, immune checkpoints, miscarriage, pregnancy loss, PD-1, RSA, recurrent spontaneous abortion, TIM-3, TIGIT, VISTA, CTLA-4

## Abstract

Immune checkpoints (ICPs) serve as regulatory switches on immune-competent cells. Soluble ICPs consist of fragments derived from ICP molecules typically located on cell membranes. Research has demonstrated that they perform similar functions to their membrane-bound counterparts but are directly present in the bloodstream. Effective control of the maternal immune system is vital for a successful pregnancy due to genetic differences between the mother and fetus. Abnormalities in the immune response are widely acknowledged as the primary cause of spontaneous abortions. In our research, we introduce a novel approach to understanding the immune-mediated mechanisms underlying recurrent miscarriages and explore new possibilities for diagnosing and preventing pregnancy loss. The female participants in the study were divided into three groups: RSA (recurrent spontaneous abortion), pregnant, and non-pregnant women. The analysis of soluble forms of immune checkpoints and their ligands in the serum of the study groups was conducted using the Luminex method Statistically significant differences in the concentrations of (ICPs) were observed between physiological pregnancies and the RSA group. Among patients with RSA, we noted reduced concentrations of sGalectin-9, sTIM-3, and sCD155, along with elevated concentrations of LAG-3, sCD80, and sCD86 ICPs, in comparison to physiological pregnancies. Our study indicates that sGalectin-9, TIM-3, sLAG-3, sCD80, sCD86, sVISTA, sNectin-2, and sCD155 could potentially serve as biological markers of a healthy, physiological pregnancy. These findings suggest that changes in the concentrations of soluble immune checkpoints may have the potential to act as markers for early pregnancy loss.

## 1. Introduction

Early pregnancy loss is a significant medical event that inflicts both physical and psychological trauma on young women and their families. While most spontaneous abortions result from genetic malformations of the embryo, a substantial number can be attributed to immunological disturbances at the feto-maternal interface [1]. To achieve a successful pregnancy, it is imperative to maintain immunological homeostasis between the mother and fetus, who carries paternal antigens, and to facilitate physiological trophoblast invasion [1,2]. Dysfunctional regulation of maternal–fetal immunity has been linked to pregnancy loss [3,4]. Currently, ESHRE defines recurrent spontaneous abortion (RSA) as more than two miscarriages before 20 weeks of pregnancy [4]. Pregnancy loss can occur in the first or subsequent pregnancies. Beyond well-established causes of RSA, such as hormonal dysfunctions, chromosomal abnormalities, thrombophilic factors, and uterine anatomical malformations, approximately half of RSA cases remain of unknown etiology. Recent studies have associated RSA with maternal immunological responses to paternal antigens [5]. In this field, many questions remain unanswered.

Immune checkpoints (ICPs) play a crucial role in maintaining the balance of immunocompetent cell functions. ICPs are molecules responsible for the regulation of the activity of various immune cells, including leukocytes. The molecules that inhibit the immune system include PD-1/PD-L1, CTLA-4, TIM-3, VISTA, TIGIT, and LAG-3. The molecules, upon binding with their ligands, send inhibitory signals to the cells, resulting in reduced activity. Activation of such ICPs can lead to the transition of the cell into an anergic state or trigger the apoptotic pathway. Proper functioning of the inhibitory molecules safeguards the organism against an excessive immune response to pathogens or prevents the development of autoimmunity. Similar regulatory mechanisms are activated during physiological pregnancy [1,2,3,4,5].

**Figure 1:**
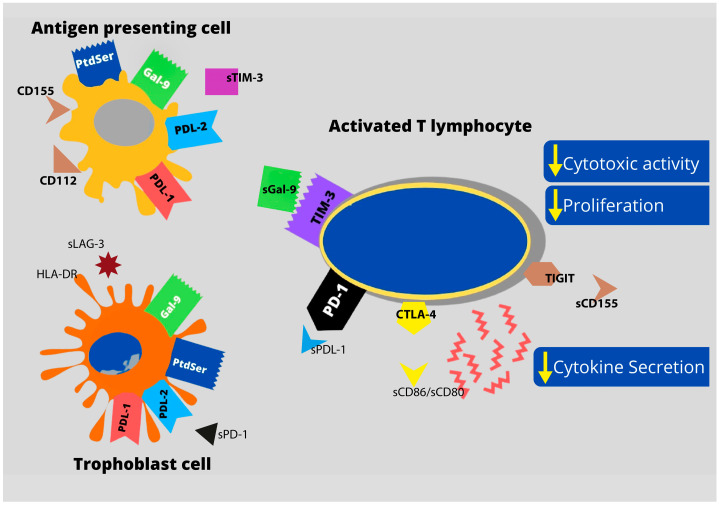
Relationship between antigen-presenting cell (APC), lymphocyte T, and trophoblast cells regulated by secreted immune checkpoints. The figure illustrates the intricate interplay among antigen-presenting cells (APC), T lymphocytes, and trophoblast cells, highlighting the impact of secreted immune checkpoints. The secretion of soluble immune checkpoints, including sPD-1, sCD80/86, sGal-9, sCD112, sCD155, etc., is depicted. Elevated soluble factors may lead to the T cell inactivation and downregulation of trophoblast antigen presentation by APC cells. The interaction of Gal-9 (Galectin-9) and PtdSer (phosphatidylserine) is crucial during implantation process. The figure is adapted from the work of Zych et al. (2021) [8], exploring differences in immune checkpoint expression (TIM-3 and PD-1) on T cells in women with RSA.

In the realm of cancer research, substantial attention has been directed toward immune checkpoint molecules such as PD-1/PD-L1, CTLA-4, TIM-3, VISTA, TIGIT, and LAG-3. The molecules play pivotal roles in activation of effector T cells, maintaining immune system homeostasis, and minimizing detrimental immune responses. Notably, tumor cells exploit immune checkpoint pathways as a mechanism for immune evasion, allowing them to elude immune surveillance—a phenomenon that appears to mirror fetal behavior [6]. Nevertheless, to date, the precise mechanisms governing immunological tolerance toward semi-allogenic fetuses remain elusive. The immunological interplay between the maternal immune system and fetal cells has yet to be comprehensively investigated [2]. The latest advancements in immunotherapy have demonstrated that manipulating immune checkpoint proteins (ICPs) can modify immune responses, either by reversing immune suppression in cancer or inhibiting cell activation in autoimmune diseases [7]. The interaction of sICPs with their ligands was pictured on Figure 1. 

Our study was grounded on the hypothesis that a comparable mechanism is in operation during normal pregnancies, and disturbances in the regulation of immune checkpoint proteins (ICPs), membrane bound or soluble, could potentially play a role in spontaneous abortions. As a result, it is conceivable that tailored antibodies could be developed to identify differences in the concentrations of soluble ICPs and their ligands, which may provide valuable insights into the significance of ICPs and their regulatory role in pregnancy. This knowledge could potentially contribute to progress in the diagnosis and treatment of pregnancy losses in the future. Furthermore, soluble isoforms of immune checkpoint proteins (ICPs) can be detected in blood samples, rendering them potential candidates as a biomarker of pregnancy loss.

## 2. Results

### 2.1. Questionnaire

Data obtained from the analysis of the questionnaire conducted among women classified for the study are presented in Table 1.

No significant differences in age or body mass index (BMI) were observed between the groups, as shown in Table 1. Patients underwent a thorough assessment, which included the number of miscarriages prior to the study, full-term pregnancies, internal medicine interviews, e.g., of chronic diseases such as diabetes, endometriosis, insulin resistance, Hashimoto’s disease, and polycystic ovary syndrome (Table 1; for detailed information, see Supplementary Data Figures S1 and S2), drug administration before and during pregnancy, and the administration of folic acid before pregnancy (Table 1; for detailed information, see Supplementary Data Figures S4 and S5). Additionally, data regarding prodromal symptoms were collected (for detailed information, see Supplementary Data Figure S3).

Among the studied groups, one non-pregnant multiparous woman and two women in the RSA group received treatment with Euthyrox, whereas four pregnant women with physiological pregnancies were in a euthyroid state; thus, participants due to active autoimmunology disease were excluded from the study.

The results shown below concern 9 non-pregnant multiparous women, 16 pregnant women, and 18 RSA.

### 2.2. Analysis of Soluble Immune Checkpoints and Ligands

**Figure 2:**
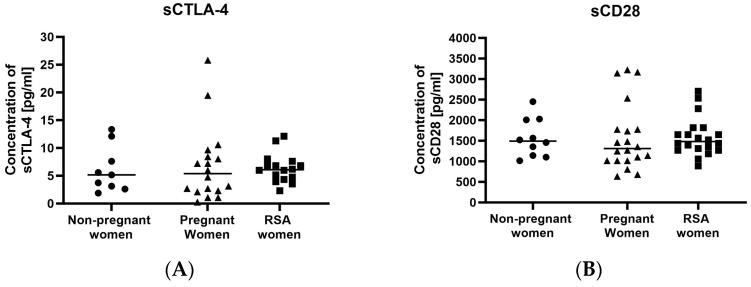

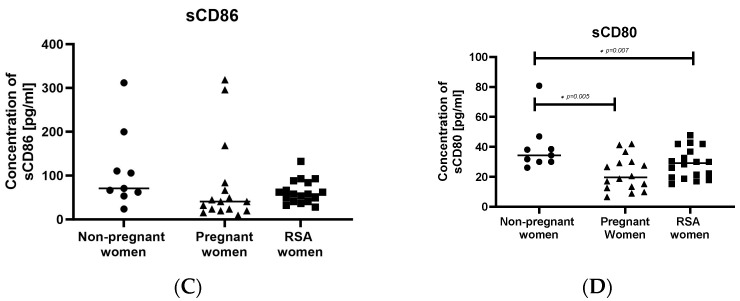
Concentrations of secretory molecules controlling the immune system (ICPs) and their ligands, (**A**) sCTLA-4, (**B**) sCD28, (**C**) sCD86, (**D**) sCD80 in the sera of studied groups of women: Group of RSA women (*n* = 18), group of pregnant women (RSA) (*n* = 16), group of non-pregnant women (*n* = 9). Results are presented as individual data points, with the mean value indicated as a line. Significance was calculated using Student’s *t*-test or Mann–Whitney U test, * *p* < 0.05.

The conducted studies did not reveal differences in the concentrations of secretory sCTLA-4 (Figure 2A) and sCD28 (Figure 2B) molecules between the studied groups. However, we observed significantly higher concentrations of sCD80 (Figure 2D) in the RSA women compared to pregnant women.

**Figure 3:**
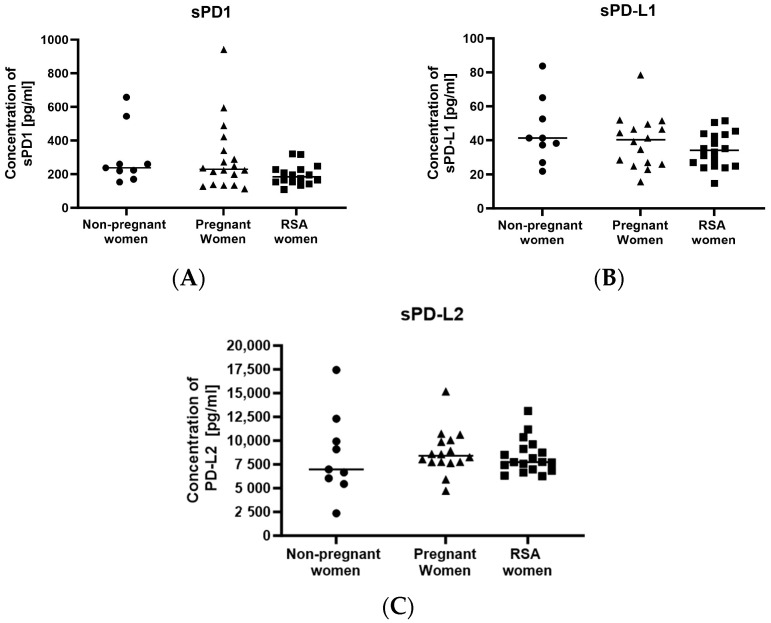
Concentrations of secretory molecules controlling the immune system: (**A**) sPD-1, (**B**) sPD-L1, (**C**) sPD-L2 in the sera of studied groups: Group of women with miscarriages (RSA) (*n* = 18), group of pregnant women (*n* = 16), group of non-pregnant women (*n* = 9). Results are presented as individual data points, with the mean value indicated as a line. Significance was calculated using Student’s *t*-test or Mann–Whitney U test.

No differences were observed between the groups in the concentrations of the sPD1 molecule (Figure 3A) and its ligands, sPD-L1 (Figure 3B) and sPD-L2 (Figure 3C).

**Figure 4:**
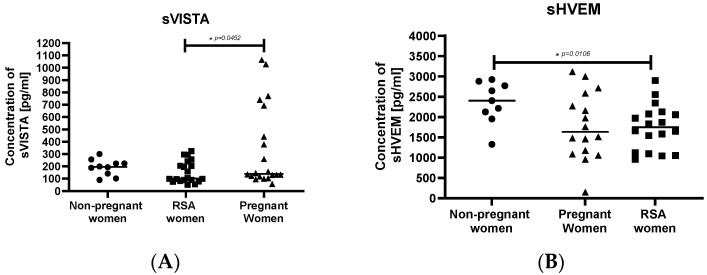
Concentrations of secretory molecules controlling the immune system, (**A**) sVISTA, (**B**) sHVEM in the sera of studied groups: Group of women with miscarriages (RSA) (*n* = 18), group of pregnant women (*n* = 16), group of non-pregnant women (*n* = 9). Results are presented as individual data points, with the mean value indicated as a line. Significance was calculated using Student’s *t*-test or Mann–Whitney U test, * *p* < 0.05.

The concentration of sVISTA was significantly higher in pregnant women compared to women with RSA (Figure 4A). Additionally, the concentration of sHVEM was higher in the non-pregnant women’s group compared to the RSA group (Figure 4B).

**Figure 5:**
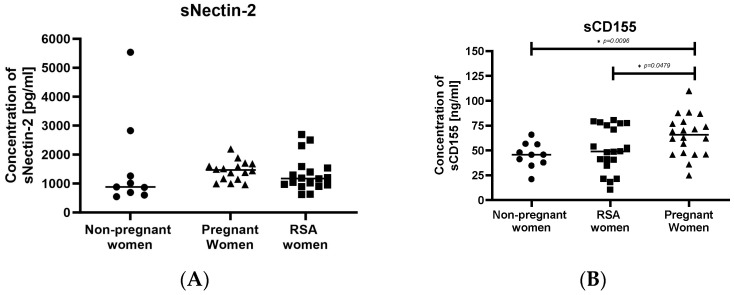
Concentrations of secretory ligands. (**A**) sNectin2, (**B**) sCD155 in the sera of studied groups: Group of women with miscarriages (RSA) (*n* = 18), group of pregnant women (*n* = 16), group of non-pregnant women (*n* = 9). Significance was calculated using Student’s *t*-test or Mann–Whitney U test, * *p* < 0.05.

The concentrations of soluble ligands of the TIGIT molecules, sNectin-2 (sCD112) and sCD155, are shown in Figure 5. SCD155 was lower in RSA women compared to pregnant women (Figure 5B). Pregnant women exhibited the highest concentration of sCD155 in comparison to the other groups (Figure 5B).

**Figure 6:**
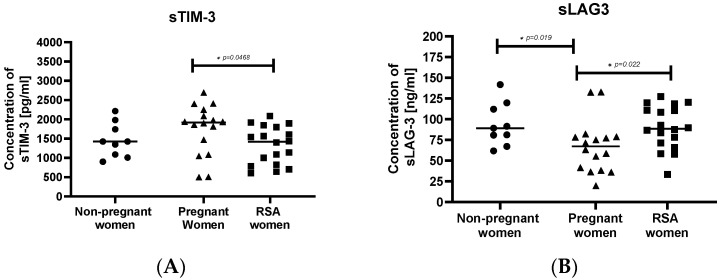
Concentrations of secretory molecules controlling the immune system. (**A**) sTIM-3, (**B**) sLAG-3, in the sera of studied groups: Group of women with miscarriages (RSA) (*n* = 18), group of pregnant women (*n* = 16), group of non-pregnant women (*n* = 9). Results are presented as individual data points with the mean value indicated as a line. Significance was calculated using Student’s *t*-test or Mann–Whitney U test, * *p* < 0.05.

The concentration of sTIM-3 was significantly higher in the pregnant women group compared to the RSA group (Figure 6A). Furthermore, pregnant women exhibited the lowest concentration of sLAG-3 among the studied groups (Figure 6B).

**Figure 7:**
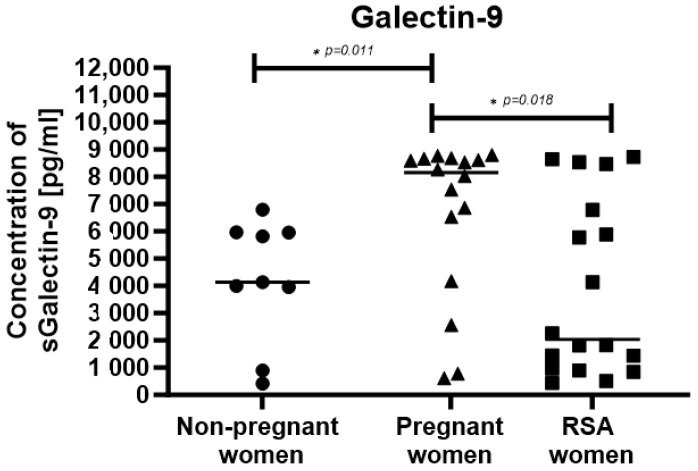
Soluble Galectin-9 concentration in studied groups: Group of women with miscarriages—RSA (*n* = 18), group of pregnant women (*n* = 16), group of non-pregnant women (*n* = 9). Results are presented as individual data points with the mean value indicated as a line. Significance was calculated using Student’s *t*-test or Mann–Whitney U test, * *p* < 0.05. The statistically significant differences marked with lines.

The concentration of the soluble sGal-9 molecule was the highest in pregnant women compared to the other studied groups (Figure 7).

Our analysis revealed that women with RSA miscarriages had rather similar levels of sICPs and ICP ligands to non-pregnant women, with an accompanying decrease of sHVEM and sGalectin-9, compared to pregnant women, decreased concentrations of sGalectin-9, sTIM-3, sCD155, and sVISTA. and increased concentrations of sLAG 3, and sCD80. Table 2 summarizes our observations.

## 3. Discussion

The most well-documented forms of immune checkpoint proteins (ICPs) are the membrane-bound variants. Nonetheless, numerous scientific publications have detailed soluble ICPs and their associated ligands. The molecules hold a pivotal role in the regulation of immune responses, contribute significantly to the development and prognosis of immune response (Figure 1), and serve as potential biomarkers and targets for emerging immunotherapies [9].

Elevated concentrations of soluble CTLA-4 (sCTLA-4) have been reported by Gu et al. in patients with breast cancer [10]. Omura et al. discovered that sCTLA-4 and soluble PD-L1 (sPD-L1) may have prognostic implications for patients with colorectal cancer [11]. The research of Wang et al. revealed elevated levels of soluble CD28 (sCD28) and decreased sCTLA-4 levels in the plasma of patients with neuromyelitis optica and multiple sclerosis [12]. Other studies have shown that soluble LAG3 (sLAG3) and sCD28 were negatively correlated with the cytolytic activity of T cells in clear-cell renal cancer [13]. Cao et al. determined that sCD28 and sCTLA-4 were elevated in patients with chronic HBV infection [14]. However, there is a limited body of knowledge regarding the concentrations of sCTLA-4 and sCD28 during pregnancy or in cases of pregnancy loss. Merely, Misra et al. have established a link between reduced sCTLA-4 secretion and the statistically significantly higher occurrence of minor allele homozygous rs231775 and rs3087243 tag-SNPs in RSA cases [15]. Our results are contradictory; the levels of sCTLA-4 and sCD28 were comparable among women with recurrent spontaneous abortion (RSA), pregnant women, and non-pregnant women.

Ip et al. concluded that the extent of changes in the concentrations of sCTLA-4, sCD28, sCD86, and sCD80 in plasma may correlate with the severity of acute asthma [16]. We found increased concentrations of sCD80 in the sera of RSA patients compared to pregnant women. CD80 binds as a ligand to the costimulatory molecule CD28 on the surface of naïve T cells and to the inhibitory receptor CTLA-4 expressed on activated T cells [16]. Soluble forms of the mentioned proteins may act similarly to their membrane-bound counterparts, either activating or inhibiting activated T cells. However, it is important to note that CD80 and CD86 have a higher binding affinity to CTLA-4 than CD28. Consequently, we can speculate that the elevated concentrations of sCD80 in RSA women may lead to an excessive suppression of activated T cells. This, in turn, could result in immunological disruptions at the feto-maternal interface during, e.g., embryo implantation, when inflammation is required [5]. The determination of sCD80 and/or sCD86 were utilized as a marker of poor prognosis for inflammatory conditions like rheumatoid arthritis or hematological malignancies [17,18].

Subsequently studied ICPs were soluble T cell immunoglobulin domain and mucin domain 3 (TIM3). TIM3 was initially identified as an inhibitory molecule in IFNγ-producing T cells. Numerous cell types, including regulatory T cells (T_reg_ cells), myeloid cells, natural killer (NK) cells, and mast cells have been shown to express TIM-3 [19,20]. In the studies involving pregnant and preeclamptic women, the pivotal role of sTim-3 was reaffirmed. Li et al. emphasized the significance of TIM-3-expressing NK cells, and that the interaction between TIM-3 and Gal-9 led to the activation of IL-10 and TGF-β genes, thus enhancing the generation of Treg cells [21]. Grossman et al. found a positive correlation between sTIM-3 levels and TNF-α, HSP70, and Gal-9 in the serum of pregnant women. Furthermore, sTIM-3 level was positively correlated with the gestational age at delivery [22]. In line with the aforementioned findings, we observed an elevation of the sTIM-3 level in the serum of pregnant women. However, Wu et al. reported increased sTIM-3 and Galectin-9 concentrations in the sera of RSA patients [23]. In our study, healthy pregnant women exhibited the highest sTIM-3 and Gal-9 concentrations in the serum. The discordant data may be attributed, in part, to differences in the group sizes of the tested RSA patients (*n* = 35 vs. *n* = 18). Nevertheless, as noted by Meggyes et al., the engagement of TIM-3 with its ligand Gal-9 leads to the apoptosis of Th1 and Th17 cells [24]. Consequently, heightened expression of TIM-3 and its shedding may influence positive pregnancy outcomes.

Furthermore, Meggyes et al. discovered that during a healthy pregnancy, soluble Galectin-9 concentration increased progressively with each trimester [24]. In line with the findings of Meggyes et al., our research demonstrated an increase in the concentration of sGalectin-9 in pregnant women’s serum if compared to non-pregnant or RSA women. Enninga et al. extended their assessment of sGal-9 by including additional time points and showed that maternal blood levels of sGal-9 remained elevated throughout gestation [25]. Both Meggyes and Enninga’s studies showed that concentrations of both soluble Galectin-9 and sPD-L1 increased during pregnancy [24,25]. However, we did not find an elevation of sPD-L1 concentration in pregnant women or in RSA serum. It is worth noting that the placenta exhibits a tremendous expression of Gal-9 and PD-L1, which might be associated with appropriate placental development throughout pregnancy [26].

Hadley et al. proved that sPD1 concentration correlates with the active disease state of autoimmune hepatitis and inflammatory bowel disease in pediatric patients [27,28]. Zhou et al. showed that serum sPD-1 levels correlate with numerous clinical parameters, reflecting inflammation and viral replication in patients affected by the chronic hepatitis B virus. The authors suggested that sPD-1 may serve as a new biomarker of liver fibrosis and can further aid in selecting antiviral treatment [27,28]. Similarly, Chang et al. found that sPD-1 and sPD-L1 may serve as prognostic markers in the progression of hepatocellular carcinoma [28,29]. Concerning pregnancy research, Gu et al. showed that maternal sPD-1 levels were significantly higher and PD-L1 relatively higher in preeclamptic than in normotensive pregnant women [30]. The authors conclude that aberrant crosstalk between sPD-1 and sPD-L1 signaling is characteristic in preeclampsia. Moreover, elevated maternal sPD-1 and sPD-L1 concentrations were associated with fetal gender differences and immune tolerance distinctions during pregnancy [30]. sPD-L1 has been shown to be a potential discriminatory marker for endometriosis-related infertility [30]. However, Okuyama et al.’s research indicated that sPD-L1 levels are elevated in the third trimester of pregnancy when compared to non-pregnant individuals [31]. In our findings, we do not find differences between groups in terms of sPD-1, sPD-L1, and sPD-L2 concentrations. It is important to clarify that our study did not include preeclampsia or endometriosis patients, cases where we found an abundance of literature. The timing of studies, the timing of data collection, and the specific populations studied could play a significant role in the observed discrepancies in the collected data. Further research on a larger scale might help to clarify and reconcile the irregularities and discrepancies [30].

Studies related to the herpes virus entry mediator (HVEM), sHVEM, or mHVEM in pregnant women or pregnancy diseases are limited. HVEM is a receptor for LIGHT, a tumor necrosis factor (TNF) superfamily ligand. LIGHT has emerged as a potent initiator of the T cell costimulation signal effecting CTL-mediated tumor rejection, allograft rejection, and graft versus host disease [32]. Gill et al. found that HVEM was present in syncytiotrophoblast and amnion epithelial cells, but it was absent in villous mesenchymal cells and cytotrophoblasts [32]. Wang et al. investigated the role of the LIGHT vs. HVEM relation in pregnancy and pregnancy-related disorders, particularly preeclampsia [33]. The research revealed that elevated LIGHT levels, coupled with heightened HVEM receptor activation, cause placental damage and the release of potent vasoactive factors such as soluble fms-like tyrosine kinase-1 (sFlt-1) and endothelin-1 (ET-1) during pregnancy [33]. The findings strongly imply that LIGHT signaling might be a pivotal factor in the development of preeclampsia [33]. Our results are contradictory; the sHVEM level decreased during pregnancy, with the lowest concentrations noted in patients who experienced miscarriage. Generally, it has been shown that sHVEM levels are upregulated in the serum of patients suffering from allergic asthma, atopic dermatitis, rheumatoid arthritis, and various neoplastic diseases [34,35].

Among others, the next studied sICP was LAG-3, also known as lymphocyte-activation gene 3, which is a protein encoded by the LAG3 gene in humans. LAG-3 is a type I transmembrane protein with structural similarities to CD4, and it is expressed 3–4 days post-activation on both CD4 and CD8 T cells [36]. In addition, LAG3 expression was found on activated T cells, NK cells, B cells, and plasmacytoid dendritic cells [37]. The molecule binds a non-holomorphic region of major histocompatibility complex class II (MHC class II) [38]. LAG-3 utilizes an additional 30 amino acid loop in the D1 region, which binds to MHC class II with greater affinity than CD4 [37,38].

Ching-Tai Huang’s research suggests that LAG-3 plays a crucial role in the function of natural and induced regulatory T cells (Tregs). The discovery supports the conclusion that LAG-3 is an essential receptor for Tregs, which plays a crucial role in pregnancy tolerance development [39]. Recent research conducted by Marozio et al. on endometrial biopsies from RSA women with dysfunctional uterine bleeding and previous uneventful pregnancies as controls showed intensified expression of genes and proteins of CTLA-4 and LAG-3 in the endometrial tissue of RSA women [40]. The results are in line with ours considering the sLAG-3 concentration in RSA women’s serum samples.

An additional immune checkpoint protein (ICP) studied by us was VISTA (B7-H1), a negative checkpoint regulator (NCR). VISTA is primarily expressed on various immune cells, including T cells, myeloid cells, and dendritic cells. The VISTA function is multifarious and evokes inhibitory and stimulatory effects on immune responses, depending on the context [41]. VISTA shares significant homology with PD-L1 and PD-L2 [41]. Wu et al. established that serum VISTA could serve as a potential novel biomarker in pancreatic cancer diagnosis [42]. We noticed that serum RSA women exhibit significantly lower concentrations of sVISTA than pregnant women.

We aimed to evaluate the concentration of ligand for TIGIT, which is the soluble form of Nectin-2 (CD112). TIGIT could be engaged with the two ligands, CD155 (PVR) and CD112 (PVRL2, Nectin-2), expressed by tumor cells and antigen-presenting cells in the tumor microenvironment. There is substantial evidence demonstrated in vivo and in vitro that the TIGIT pathway plays a role in T-cell-mediated and natural killer cell-mediated tumor recognition. Dual blockade of PD-1 and TIGIT has been shown to significantly enhance the expansion and function of tumor antigen-specific CD8^+^ T cells in vitro and promote tumor regression in mouse tumor models [43]. Nevertheless, in the existing literature, we have not encountered examples of utilizing sNectin for diagnostic or therapeutic purposes. Meggyes et al. evaluated the TIGIT-CD226-CD112-CD155 immune checkpoint network during a healthy pregnancy. The difference in CD226 expression and concentration among all the studied parameters was found [43].

The second ligand for TIGIT is CD155 [44]. Iguchi-Manaka et al. found that sCD155 was increased in patients with various cancer types, including esophageal, colorectal, pancreatic, bile-duct, breast, gastric, ovarian, endometrial, lung, and cervical cancers; thus, authors concluded that it might be a useful biomarker for cancer development [44]. In a separate study, Iguchi-Manaka et al. established that sCD155 concentration in the serum of patients with breast cancer was positively correlated with the patient’s age, stage of disease, and size of the invasive tumor [45]. Okumura et al. showed that sCD155 derived from tumors hinders the DNAM-1-mediated antitumor activity of NK cells [46]. This phenomenon suggests that an elevated concentration of sCD155 may lead to the downregulation of NK cell activity, e.g., during physiological pregnancy, which supports our observation of the decreased level of CD155 in RSA women.

## 4. Material and Method

### 4.1. Institutional Review Board Statement

The study received ethical approval from the Bioethics Committee of the Medical University of Warsaw (Approval Number: KB/13/2020 issued 13 January 2019). Informed consent was obtained from all participants before conducting measurements, interventions, and blood collections. The procedures adhered to the principles outlined in the Helsinki Declaration of 1975, as revised in 2013.

### 4.2. Study Groups

The study participants were categorized into three groups: RSA (recurrent spontaneous abortion) women, pregnant women, and non-pregnant women. Data were collected from all participants, including information on age, weight, height, history of early and late miscarriages, and prodromal pregnancy symptoms (e.g., vomiting, nausea, and breast pain). Additionally, we recorded details about medical procedures undertaken before and during pregnancy, the use of vitamins or dietary supplements, pre-pregnancy folic acid intake, hormonal contraception history, and previous in vitro treatments. Chronic medical conditions such as diabetes, endometriosis, insulin resistance, Hashimoto’s disease, and polycystic ovary syndrome (PCOS) were also documented. The data collection period spanned from 2019 to 2021, and all necessary precautions related to the COVID-19 pandemic were undertaken.

#### 4.2.1. Control Group

(a) Non-Pregnant Women

The non-pregnant control group consisted of 10 fertile, non-pregnant women with no prior obstetric-gynecological or internal medicine disorders—multiparous women. All the women in this group had previously given birth at least once without any complications, and they reported having experienced healthy pregnancies. None of the participants in the control group had a history of miscarriages. Additionally, apart from one individual, who had Hashimoto’s disease but was in a euthyroid state, none of the control subjects had received treatment for chronic illnesses. Blood samples were collected during the follicular phase of the menstrual cycle.

(b) Pregnant Women

Twenty pregnant women between 11–13 week of pregnancy were classified as the control “pregnancy group”. To confirm the physiological development of pregnancy, patients underwent an ultrasonographic scan following the guidelines of the Fetal Medicine Foundation. Additionally, physical examinations and blood tests mentioned above were conducted.

#### 4.2.2. Patients with Recurrent Spontaneous Abortion (RSA)

Twenty recurrent spontaneous abortion (RSA) patients diagnosed, according to ESHRE, as experiencing two or more consecutive spontaneous miscarriages before the 20th week of gestation [47,48], were recruited as the study group. The samples were collected within 72 h following the miscarriage.

### 4.3. Methods

#### 4.3.1. Sample Preparation

The blood samples were vested into 5 mL BD Vacutainer Plus Serum Tubes. After 30 min of blood collection, serum was separated from RBC by centrifugation at 2500 rpm for 20 min. One milliliter of serum was collected in 5 tubes with 200 µL volume sample in freezing tubes (MERCK, Darmstadt, Germany) and frozen at −80 °C.

#### 4.3.2. Luminex Acquisition

The concentrations of 13 ICPs were measured on MAGPIX (MERCK, Darmstadt, Germany) with a Luminex-based bead array, the MILLIPLEX^®^ MAP Human Immuno-Oncology Checkpoint Protein Panel 1 (MERCK, Darmstadt, Germany), and the MILLI-PLEX^®^ MAP Human Immuno-Oncology Checkpoint Protein Panel 2 (MERCK, Darmstadt, Germany) on the Luminex xMAP^®^ platform using a magnetic bead format (MILLIPLEX^®^ Analytes, Millipore, MA, USA) for the following biomarkers: sTIM-3, sCTLA-4, sCD80, sCD86, sCD28, sPD-1, sPD-L1, sPD-L2, sHVEM, sCD112, sCD155, sLAG-3, and sVISTA (B7-H5). For each sample, 25 µL of serum was used to assess the concentration of soluble ICPs and their ligands. All procedures were performed according to the manufacturer’s recommendations. Quality assurance was maintained through the inclusion of appropriate standards and quality controls provided in the kits. Each run incorporated relevant quality controls, and results were calculated using the xPONENT system (MERCK, Darmstadt, Germany). For specific data regarding the upper limit of quantification (ULOQ) and lower limit of quantification (LLOQ), please refer to Supplementary Materials, Table S1.

#### 4.3.3. ELISA Method

ELISA assays were conducted using commercially available reagent kits following the manufacturer’s instructions. Serum samples underwent a two-fold dilution. Galectin-9 levels were analyzed with the Human Galectin-9 ELISA Kit (Thermo Fischer, Waltham, MA, USA), and the concentrations were determined using the four-parameter logistic curve, as per the manufacturer’s instructions. The ELISA kit exhibited a sensitivity of 36.86 pg/mL.

### 4.4. Statistical Analysis

Statistical analyses were performed using GraphPad Prism 8.4.1. The results were presented as the mean ± standard deviation (SD). Gaussian distribution was assessed using the Shapiro–Wilk test. For data sets exhibiting a Gaussian distribution, statistical comparisons were made using the F test to assess equal variance, followed by unpaired *t*-tests for data sets with equal SD and unpaired *t*-tests with Welch’s correction for data sets with different SD. In cases where the data did not follow a Gaussian distribution, the Mann–Whitney U test was applied. Statistically significant differences between groups, indicated by *p*-values below 0.05, were denoted with asterisks.

## 5. Conclusions

The collected results suggest that alterations in the concentrations of certain immune checkpoint proteins (ICPs) may be associated with pregnancy loss. Specifically, we observed that women experiencing recurrent pregnancy abortions (RSA), when compared to both control groups (pregnant women and non-pregnant), exhibited decreased concentrations of sGalectin-9, sCD155, and sTIM-3. Additionally, increased secretion of sLAG-3 and sCD80 accompanies this phenomenon. The pattern of expressed sICPs by pregnant women could be correlated with the function of Galectin-9, CD155, and TIM-3, which downregulate NK and T cell activation. Whereas, overexpression of sLAG-3 may indicate trophoblast HLA antigen recognition, and increased sCD80 costimulatory molecule secretion may suggest triggering of T cell responses in RSA women.

Generally, in our research, RSA women exhibited analogous expression of soluble immune checkpoints as non-pregnant women. Considering all the compiled data, we can suggest that changes in the secretion of the following immune checkpoint proteins (ICPs) could be a potential marker for recurrent pregnancy loss: sCD155, LAG-3, Gal-9, and sTIM3, with an accompanying increase in the T-cell receptor (TCR) costimulatory molecule sCD80.

Determination of the ICPs might help to predict the fate of early pregnancy and give the possibility of introducing targeted therapy based on the observed immunological imbalance of the feto-maternal interface.

We acknowledge that the presented results should be validated in larger study populations. Given the intricate nature of the immune system’s functioning, the findings from our research catalyze further advancement in scientific research on the subject of recurrent miscarriages.

## Figures and Tables

**Table 1:** Data from the questionnaire completed by the participants in the study. Number of participants in the group: Non-pregnant multiparous women (*n* = 10), pregnant (*n* = 20), RSA (*n* = 20), *p*-values statistically significant below 0.05 (*p* < 0.05) were marked as: *, if *p* < 0.001 was marked as **; N/A: not applicable. ^1^ RSA vs. Pregnant, ^2^ RSA vs. Multiparous, ^3^ Multiparous vs. Pregnant Data are presented as median and 25th (Q1)–75th (Q4) percentile, or percentage of the group.

	Median and Q1–Q4 Quartile	Non-Pregnant Multiparous Women	Pregnant Women	RSA Women	*p*-Value ^1^ RSA vs. Pregnant ^2^ RSA vs. Multiparous ^3^ Multiparous vs. Pregnant
Age	Median	33.5	30	34	^1^ *p* = 0.26
	Q1	26	25	22	^2^ *p* = 0.26
	Q4	40	39	40	^3^ *p* = 0.4
BMI (body mass index)	Median	25	21.6	21.8	^1^ *p* = 0.5
	Q1	18.6	16.7	17.9	^2^ *p* = 0.48
	Q4	36.2	31.4	37.2	^3^ *p* = 0.28
Number of full-term pregnancies	Median	**1**	**0**	**0**	^1^ *p* = 0.027 *
	Q1	1	0	0	^2^ *p* = 0.00001 **
	Q4	3	3	0	^3^ *p* = 0.007 *
Number of miscarriages	Median	0	0	3	^1^ *p* = 00001 **
	Q1	0	0	2	^2^ *p* = 0.0001 **
	Q4	0	0	5	^3^ *p* = 0.5
Pregnancy duration (weeks)	Median	N/A	12.8	8.8	^1^ *p* = 0.1
	Q1	N/A	11.7	4.0	^2^ N/A
	Q4	N/A	14.6	13.6	^3^ N/A
The occurrence of chronic diseases					
		Non-Pregnant multiparous women	Pregnant women	RSA women	
Diabetes		0%	10%	5%	
Endometriosis		0%	0%	10%	
Insulin resistance		0%	0%	5%	
Hashimoto disease		10%	30%	10%	
Polycystic ovary syndrome		0%	10%	0%	
Diet supplements and folic acid administration before pregnancy and during pregnancy					
Folic acid administration		60%	65%	80%	
Euthyrox or acard and dietary supplements administration before pregnancy		30%	60%	75%	
Eythyrox or acard and dietary supplements administration during pregnancy		40%	90%	75%	
No medicine and dietary supplements administration before pregnancy		70%	40%	25%	
No medicine and dietary supplements administration during pregnancy		60%	10%	25%	

**Table 2:** Analysis of differences in the concentration of soluble immune checkpoint s and ICP ligands between the RSA vs. non-pregnant, multiparous women, and pregnant women. The arrows show decrease or increase of sICP concentration.

Step of Analysis	Compared Groups	Concentration of ICPs in Serum
**I**	RSA women vs. non-pregnant multiparous women	↓ sGalectin-9, ↓ sHVEM
**II**	RSA women vs. pregnant women	↓ sGalectin-9, ↑ sLAG-3, ↑ sCD80, ↓ sCD155, ↓ sTIM-3

## Data Availability

The sICP.xlsx data used to support the findings of this study are available from the corresponding author upon request.

## Supplementary Materials

The supporting information can be downloaded at: https://www.mdpi.com/article/10.3390/ijms25010499/s1.
