# Supplementary material for: Soluble Forms of Immune Checkpoints and Their Ligands as Potential Biomarkers in the Diagnosis of Recurrent Pregnancy Loss—A Preliminary Study
Source: Int J Mol Sci. 2023 Dec 29;25(1):499. doi: 10.3390/ijms25010499 (PMC10779235; doi:10.3390/ijms25010499)
Supplement: Supplementary file 1 [file ijms-25-00499-s001.zip › ijms-2694568-supplementary.pdf]

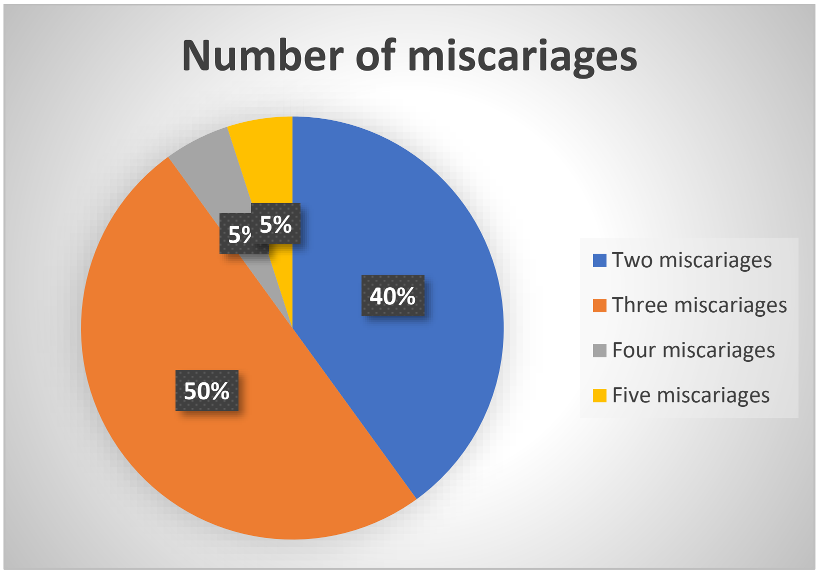

Figure S1. Precise presentation of the number of miscarriages in the RSA group n=20.

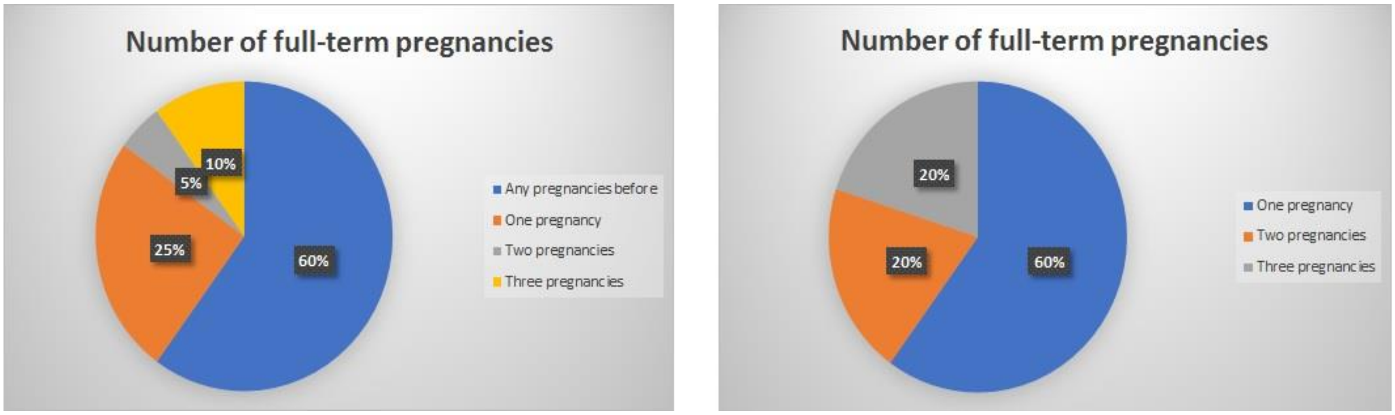

Figure S2. Precise presentation of the number of delivered pregnancies in the pregnant and non-pregnant group as percentage of the whole group, Pregnant n=20, non-pregnant n=10.

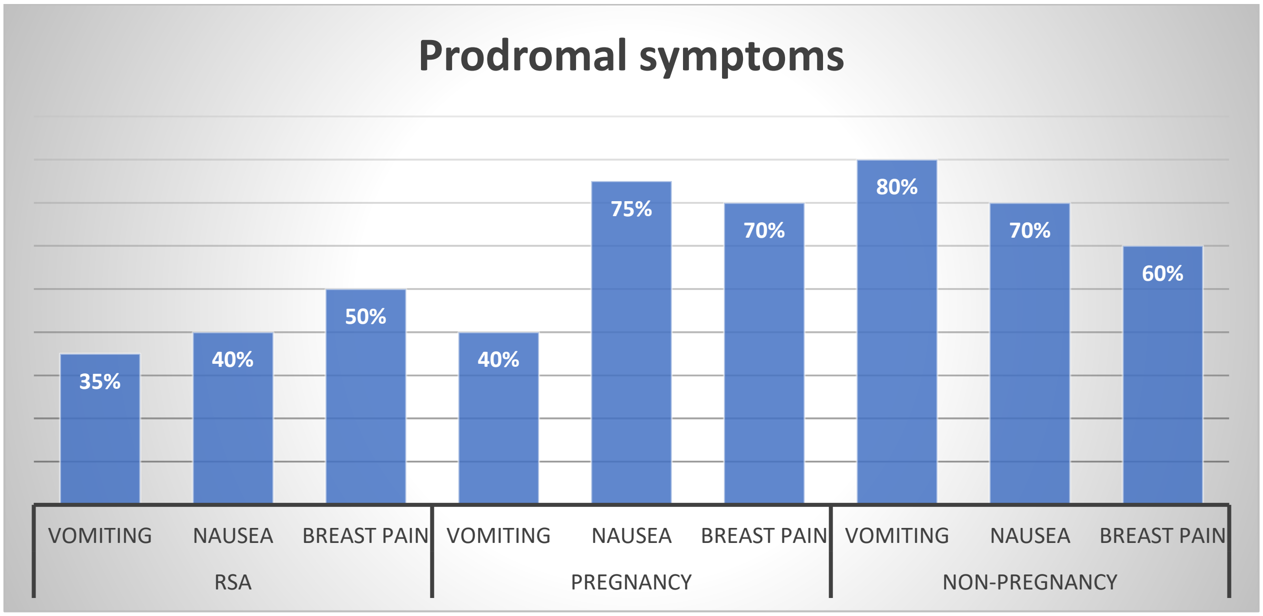

Figure S3. Rate of prodromal syndromes (vomiting, nausea, breast pain) in groups, RSA n=20, Pregnant n=20, non-pregnant n=10. Data showed as percentage.

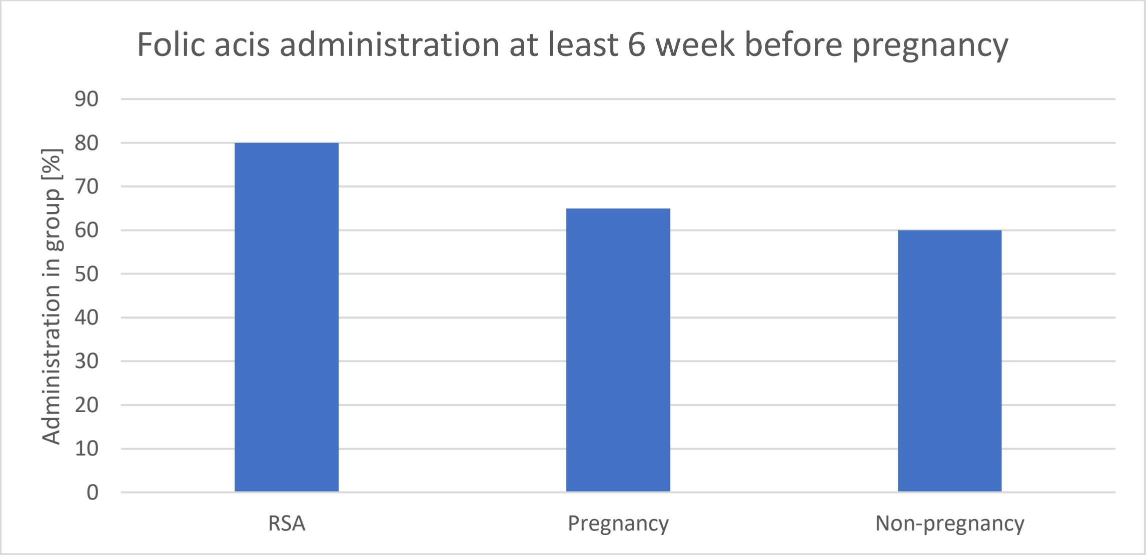

Figure S4. Folic acid administration by women in all studied group at least 6 weeks before pregnancy, RSA n=20, Pregnant n=20, non-pregnant n=10 . Data showed as percentage.

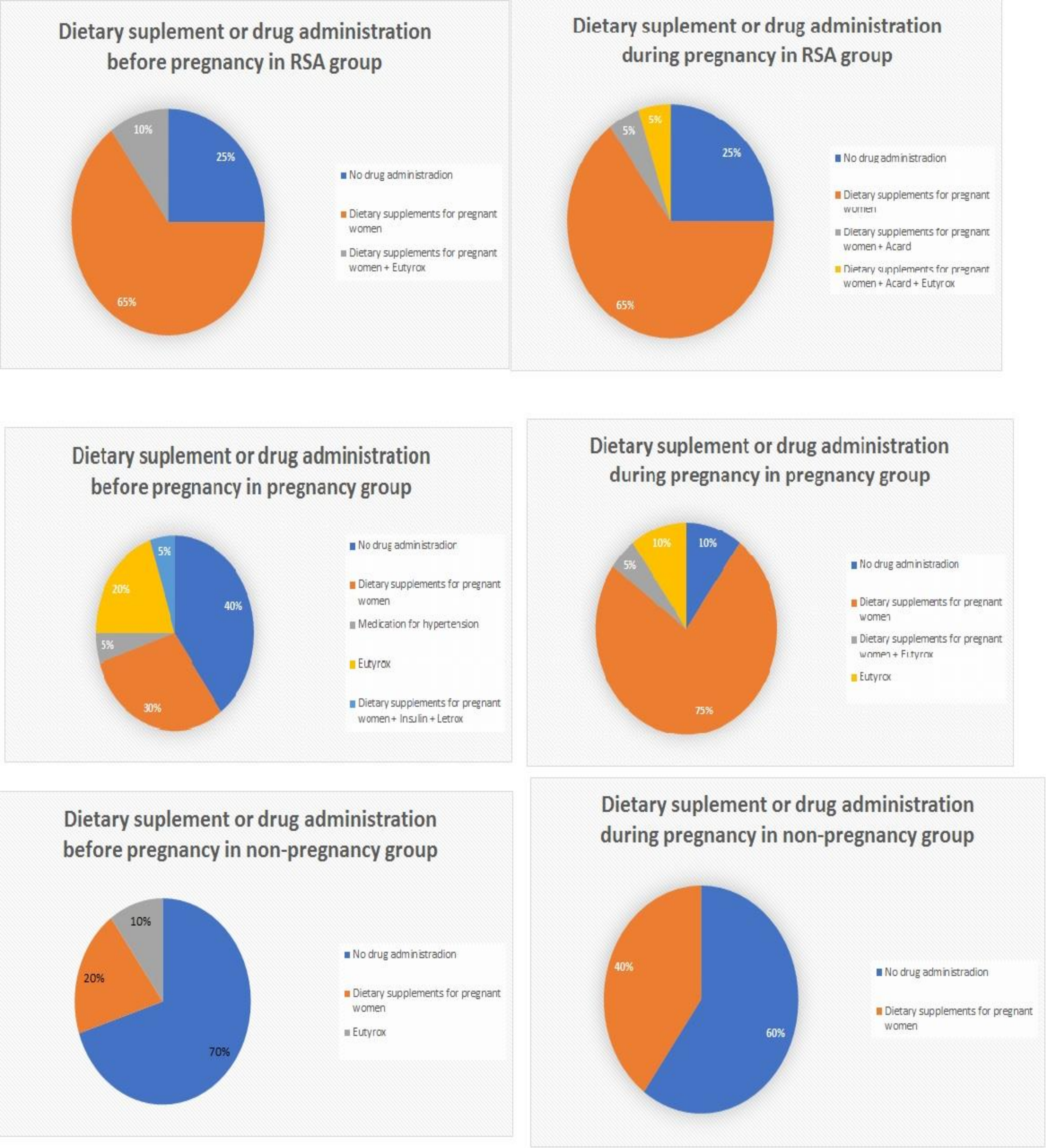

Figure S5. Drug administration by women in all studied group before and during pregnancy RSA n=20, Pregnant n=20, non-pregnant n=10. Data showed as percentage.

Tabele S1. Lower Limit of Quantification (LLOQ) and Upper Limit of Quantification (LOQ) values for tested sICPs using the Luminex device. Data showed as concentration, pg/ml.

sICP	ULOQ [pg/ml]	LLOQ [pg/ml]
sVISTA	8850 pg/ml	8,64 pg/ml
sCD28	133000 pg/ml	32 pg/ml
sCD80	17130 pg/ml	4,2 pg/ml
sLAG	50900 ng/ml	12 ng/ml
sPD-1	28600 pg/ml	6,98 pg/ml
sPD-L1	14300 pg/ml	3,49 pg/ml
sPD-L2	185400 pg/ml	45 pg/ml
sTIM-3	244100 pg/ml	60 pg/ml
sCD155	30250 ng/ml	3,45 ng/ml
sNectin-2	50225 pg/ml	49 pg/ml
sHVEM	61500 pg/ml	15 pg/ml
sCD86	2600 pg/ml	2,48 pg/ml
sCTLA-4	8775 pg/ml	1,57 pg/ml
